# Early days of life are crucial for child survival in Gamo Gofa Zone, Southern Ethiopia: A community based study

**DOI:** 10.1186/s12887-016-0568-z

**Published:** 2016-03-05

**Authors:** Girma Temam Shifa, Ahmed Ali Ahmed, Alemayehu Worku Yalew

**Affiliations:** School of Public Health, Addis Ababa University, Addis Ababa, Ethiopia

**Keywords:** Under five, Infant, Neonatal, Child, Mortality, Death, Determinants of mortality, Gamo Gofa, Ethiopia

## Abstract

**Background:**

Though, Ethiopia has shown progress in the reduction of under-five mortality in the last few years, the problem of neonatal and under-five mortality are still among the highest in the world and that warrants continuous investigation of the situation for sustained interventions to maintain the reduction beyond the millennium development goals. Therefore, this study was conducted with the objective of determining the magnitude of childhood mortalities in the designated community.

**Method:**

A census of 11 kebeles (lowest administrative units in Ethiopia) of Arba Minch Town and 11 kebeles of Arba Minch Zuria District, which were not part of Arba Minch Demographic Surveillance System (DSS), had been done in order to identify all children (alive and dead) born between September 01, 2007 and September 30, 2014. Besides, all children born after July 01, 2009 were tracked from the data base of the Arba Minch DSS. Descriptive analyses with frequency and cross tabulation with the corresponding confidence interval and p-value were made using SPSS 16 and STATA 11. Extended Mantel-Haenszel chi-square for linear trend was also performed to assess presence of linear trend through the study period using open-Epi version 2.3.

**Result:**

A total of 20,161 children were included for this analysis. The overall weighted under five, infant and neonatal mortalities with their corresponding 95 % confidence intervals were: 42.76 (39.56-45.97), 33.89 (31.03-36.76) and 18.68 (16.53-20.83) per 1000 live births, respectively. Majority of neonatal deaths occurred within the first 7 days of life. Under-five mortality was found to be significantly higher among non-DSS rural kebeles, overall rural kebeles and females.

**Conclusion:**

Significant number of children died during their early days of life. Strengthening of maternal and child health interventions during pregnancy, during and immediately after birth are recommended in order to avert majorities of neonatal deaths.

## Background

Unacceptably, every day 17,000 children die before their fifth birthday in the world, mostly from preventable and treatable causes. In actual number, only in 2013, 6.3 million children died before their fifth birth date. This is despite the existence of knowledge and technologies for life-saving interventions [1]. In 2012, about 75 % of all child deaths were attributable to just six conditions: child birth related neonatal causes, pneumonia, diarrhea, malaria, measles, and HIV/AIDS [2].

Inequities in child mortality between high income and low income countries continue to exist. For instance, in 2013 the under five-mortality rate in sub-Saharan African Region was the highest in the world, 92 deaths per 1000 live births, nearly 15 times the average in developed countries [1].

In Ethiopia, under-five mortality was reported to decline by 47 % over the 15-year period between the 2000 and the 2011 Ethiopian Demographic and Health Survey (EDHS) (declined from 166 to 88 deaths per 1000 live births). Infant mortality also decreased by 39 % over the same period, from 97 to 59 deaths per 1000 live births [3]. Although such decline has been reported, child mortality rate in Ethiopia has been among the highest in the world, that about one in every 17 Ethiopian children dies before the first birthday, and one in every 12 children dies before the fifth birthday [3].

The neonatal (37 per 1000 live births) and post-neonatal (22 per 1000 live births) mortality rates were also high in the country, where relatively slow reduction was observed. Childhood mortality in the country is higher in rural areas than in urban areas [3]. Mortalities in Southern Nations, Nationalities and People’s Region (SNNPR) were among the highest in the country. Under five, child, infant, post neonatal and neonatal mortalities in the Region were 116, 41, 78, 41 and 38 per 1000 live births, respectively [3].

Besides the periodic EDHS reports, few pocket studies which have been conducted in other parts of the country showed varying figures. Under-five mortality was ranging from 76 to 130 per 1000 live births, whereas infant mortality was ranging from 62 to 93.5 per 1000 live births [4–7]. However, these studies were basing only on few kebeles (lowest administrative units in Ethiopia) of DSS sites or they were not meant to assess the magnitude of childhood mortalities. For example, a study at the DSS site of Butajira, Ethiopia, reported an infant mortality rate of 62/1000 live births [4], though its main objective was not to assess the magnitude of infant mortality. In another DSS based study done in Dabat, Northern Ethiopia, the risk of infant death was 93.5 per 1000 live births, whereas under five mortality was 130 per 1000 live births [5]. A relatively recent study in the same DSS site (Dabat) showed infant mortality of 88 per 1000 person-years [8].

Another community based study in the northern part of the country reported, neonatal, post neonatal, infant, child and under five mortality rates of 37, 30, 67, 33 and 99 per 1000 live births respectively [6]. A study in the South West part of the country also reported neonatal and infant mortality rates of 38 and 76.4 per 1000 live births, respectively [7].

As the Arba Minch DSS (study site of the current study, which is located extreme south of the country) is new, under-five mortality studies are lacking in the area. The above mentioned studies are concentrated around central or northern part of the country that it may not be possible to have nationally representative summary of magnitude of the problem from these studies. Overall, Ethiopia has shown progress in the reduction of under-five mortality in the last few years; however, the problem is still among the highest in the world and warrants for continuous investigation of the problem for sustained interventions to maintain the reduction beyond the millennium development goals (MDGs). Therefore, this study was conducted with the objective of determining the magnitude of childhood mortalities in the designated community.

## Methods

### Study area

The study was conducted in Gamo Gofa Zone, which is one of the 14 Zones in the Southern Nations Nationalities and People’s Region (SNNPR). The Zone has 15 districts (woredas) and 2 town administrations. Arba Minch Town, the Capital of Gamo Gofa Zone, is 502 km south of Addis Ababa. Gamo Gofa Zone is a zone with two Lakes (Lake Chamo and Abaya). The Zone is known for its banana, apple and fish production which may impact child nutrition and survival. There were three hospitals and 68 health centers providing health services for the population during the study period. In 2014, the total population of the Zone was projected to be 1,901,953 (with 943,834 males and 958,119 female, 285,043 Urban (15 %) and 1,616,910 Rural (85 %) residents) [9].

Arba Minch Zuria District has been selected as study site for the current study, as it is the study site for the Arba Minch DSS which is relatively new site in the country and as the District has three climatic/geographic zones (Dega(high land), Woina dega (mid land) and Kolla(low land)); which is suitable to represent population of different agro ecological zones. The District lies on 168,712 square kilometers and constitutes 29 kebeles (lowest administrative units in Ethiopia). The total population of the district was projected to be 185,302 (with 92,680 males and 92,622 female) in 2014. Arba Minch Town, which is the capital of the Zone, is included to represent the urban population of the Zone. The total population of the Town was projected to be 135,452 (with 68,132 males and 67,320 female) [9]. The Town was divided in to 11 urban kebeles.

The Arba Minch DSS was established in 2009 in one of the districts in the Zone (Arba Minch Zuria District), which was part of the current study. Arba Minch DSS is based in 9 kebeles of the district. It was established by conducting base line survey/census during July 01-September 30, 2009. Since then, it has been tracking information on vital events (birth, death, migration etc.) continuously. The total population of the DSS was 59,875 with 12,241 female in the reproductive age (15-49), 9825 under-five and 2388 under one year of age children *(2011 report of the DSS).*

### Study design and period

A cross-sectional study design was conducted to assess the magnitude of under-five mortality in 2014, as part of a doctoral thesis work “assessment of magnitude and determinants of under-five mortality and its effect on maternal mental health in Gamo Gofa Zone, Ethiopia”.

### Source and study population

The source population was all under-five children in the study area whereas, the study population was all children born between September 01, 2007-September 30, 2014.

#### Inclusion criteria

All children (alive and dead) together with their respective mothers born between September 01, 2007-September 30, 2014 were included in the study.

#### Exclusion criteria

Those who were still births were excluded.

### Sample size determination

The sample size was determined using single population proportion formula by considering the prevalence of under-five mortality to be 88/1000 [3]. By taking 95 % confidence level and 1.5 % margin of error, the minimum required sample size for the study was 1371. By applying a design effect of 1.5 and adding 5 % to compensate for non-response, a total of 2158 under five children were required. However, all children who had been identified during census of the selected kebeles were included in the analysis to increase the precision and able to estimate other categories of childhood mortality rates.

### Sampling technique

Arba Minch Town and the Arba Minch Zuria District were selected purposively out of the 15 districts and 2 town administrations of the Zone. Then, all the 11 kebeles of Arba Minch Town and the 9 kebeles of the Arba Minch Zuria District which are part of the Arba Minch DSS were included (initially these kebeles were selected randomly out of 29 kebeles in the District) and additional 11 kebeles from those kebeles which were not part of the Arba Minch DSS were selected randomly. Accordingly, 31 kebeles from the two districts were included in this study (11 from Arba Minch Town and 21 from the Arba Minch Zuria District). This number was assumed to provide adequate number of sample for the subsequent studies.

Then, a census of the 11 non-DSS kebeles of the Arba Minch Zuria District and 11 kebeles of Arba Minch Town had been done in order to identify all children (alive and dead) born between September 01, 2007-September 30, 2014. The children were followed retrospectively by asking the respondent about whether the child was alive or dead at the time of the survey. If the child was dead, the date of death was recorded.

**Fig. 1:**
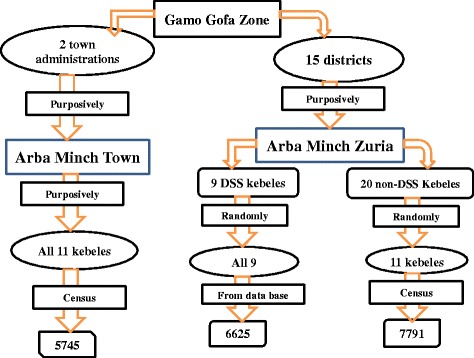
Schematic presentation of the study districts and kebeles and their selection procedure

As the Arba Minch DSS has been tracking all births and deaths since its establishment in 2009, children born between August 01, 2009 and September 30, 2014 in Arba Minch DSS kebeles were tracked from the data base of the DSS. Therefore the data since 2009 were tracked from all the 31 kebeles and the data since 2007 were tracked only from the 22 kebeles (Fig. 1).

### Data collection

A pre-tested Amharic questionnaire was utilized for data collection. The questionnaire was developed in English and translated to Amharic, then back translated to English to check for its consistency. Finally, the Amharic Version was used for data collection. Variables in the questionnaire include: sex of the child, date of birth of the child, whether the child is alive or dead, if dead date of death and other identifiers (identification number (for the child and the mother), district name, kebele name, house number). At least two data collectors (grade 10 or above) per kebele were recruited and trained on the procedure. Four master holders (in Public Health) supervised the data collection process. The principal investigator had been strictly following the data collection throughout the process. Besides, additional data were sought from the kebele admirations and health posts through reviewing documents and/or interviewing the kebele officials or the health extension workers (HEWs) to determine characteristics of the kebeles.

### Data processing and management

The data were edited, coded, entered into computer and cleaned using Epi Info Version 3.5.1 and the analysis was performed by open-epi version 2.3, SPSS version 16 and STATA 11 as appropriate. The daily collected data were transferred to the Arba Minch University and locked in a secure cabinet which was arranged in the compound of College of Health Sciences of the Arba Minch University on daily basis. The data were entered into Epi info by two data encoders after having training/orientation on the template, the procedures for insuring the quality of the data during data entry and the importance of quality of data. They were also expected to identify incomplete and inappropriate data and communicate to the principal investigator at this stage too. This was strictly followed and checked by the principal investigator on daily basis.

### Data analysis

Descriptive analyses with frequency and cross tabulation with the corresponding confidence interval with *p*-value were made. As we collected data on complete live birth histories of all mothers within the last 7 years before the survey, we have applied a direct method to estimate mortalities.

Accordingly, birth cohort method was applied to determine overall level of childhood mortalities (only deaths of children born during the study period were included in the numerator). Whereas, death cohort method was used for trend analysis (deaths of children born prior to the target year may be included in the numerator of that year). Extended Mantel-Haenszel chi square for linear trend was also performed to assess presence of linear trend through the study period using open-epi version 2.3.

Weighted analysis was conducted to account for the non-proportional allocation of the sample to urban and rural kebeles. The sampling weight was calculated using the following notion: by determining sampling probability at two stages (district and kebele levels), as a complete census/coverage of individuals in selected kebeles was made.

P(k^th^ individual in j^th^ kebele in i^th^ district being selected) = P(i^th^ district being selected)P(j^th^ kebele selected| i^th^ district is selected)P(k^th^ individual selected| j^th^ kebele is selected, which is one (as a complete census was made)). Then the weights were the reciprocals of these probabilities [10].

Accordingly, the sampling weight for urban was: As one out of 2 urban districts was included and all the kebeles in selected district were included. The probability of selection of individuals in urban kebele = 1/2*1*1 = 0.5. The corresponding weight calculated to be 2. For that of rural: as one out of 15 rural districts was included, twenty out of 29 kebeles of the district were included. The probability of selection of individual in rural = 1/15*20/29*1 = 0.046. The corresponding weight calculated to be 21.8.

### Data quality assurance

The questionnaire was pre-tested and corrections were made accordingly. Two days training was given to data collectors and supervisors on the questionnaire and the procedures. The data collection process was strictly followed up. All collected data were checked every day for their completeness, clarity and consistency by supervisors and the principal investigator. Any unclear and ambiguous data were corrected by recollecting data from actual study population by going back to the field, while minor errors were corrected by the principal investigator as deemed necessary. About 5 % of the households were re-visited by the supervisors/principal investigator to check the validity of the information collected by the data collectors. Then, data were cleaned and checked before data entry and analysis again. Besides, double entry of 10 % of the questionnaire was made to monitor any discrepancies.

### Ethical considerations

Ethical clearance and approval was obtained from the Institutional Review Board of the College of Health Sciences at Addis Ababa University. Letters were written to all concerned bodies (Gamo Gofa Zone Health Department, Arba Minch Zuria District and Arba Minch Town Health Office and administration of all kebeles) and permissions were secured at all levels. After explaining about the purpose of the study and confidentiality of the data, verbal consent was obtained from each respondent. To assure the confidentiality of the responses, anonymous interviews were conducted. Besides, the daily collected data were transferred to the Arba Minch University and locked in a secure cabinet on daily basis.

## Result

### Basic characteristics of the study subjects/kebeles

Overall, 13536 children born between September 2007 and September 2014 were identified from the census of 11 kebeles of the Arba Minch Town and 11non-DSS rural kebeles of the Arba Minch Zuria District. Additional data from 6625 children born between August 2009 and September 2014 were obtained from the Arba Minch DSS data base. A total of 20161 children were included for this analysis. Accordingly, 6625 (32.9 %), 7791 (38.6 %) and 5745 (28.5 %) of the children were from DSS sites, none DSS rural kebeles of Arba Minch Zuria District and Arba Minch Town, respectively.

Majority (27/31) of the kebeles had all-weather road. More than half (19/31) of the kebeles were more than 10killo meters (kms) away from the serving hospital in the area (Arba Minch Hospital). Except one kebele, all the 30 kebeles were within 10kms from the nearby health center. Majority (23/31) of the kebeles’ staple food was maize. Most (19/31) of the kebeles were malarious. All kebeles had at least one HEW working in the kebele during the study period. Almost all (29/31) had at least two HEWs working in the kebeles. A maximum of 4 HEWs were found in some kebeles. In about half (14/31) of the kebeles, HEWs were providing delivery service at home or in the health post during the study period.

Overall, 10,375 (51.5 %) of the children were female giving a male to female ratio of 1:1.06. Majority (14,416 (71.5 %)) of the children were from rural kebeles. Five hundred eighty five (2.9 %) of the children were neonate. Three thousand eight hundred twenty five (19 %) of the children were less than one year old. Majority (13,512 (67.0 %)) of the children were from kola (low land) kebeles. Majority (71.5 %) of the children were living more than 10kms away from Arba Minch hospital. Whereas, 95.2 % of the children were living within 10kms distance of the nearby serving health center (Table 1).

**Table 1:** Socio-economic characteristic of the study subjects/study kebeles, Gamo Gofa Zone, 2014

Characteristics	Frequency	Percent
Distance from Arba Minch Hospital in KM of the household		
<=10km	5745	28.5
>10KM	14416	71.5
Distance from Nearby Health center in KM of the household		
<=10Km	19189	95.2
>10Km	972	4.8
Sex of the child		
Male	10375	51.5
Female	9786	48.5
Age category of the child		
Neonate	585	2.9
Post Neonate	3240	16.1
Infant	3825	19
Child	16336	81
Under-five	20161	100
Kebele category of the child		
DSS-Rural	6625	32.9
Non-DSS Rural	7791	38.6
Urban	5745	28.5
Climatic/agro-ecological Zone of the child		
Kola (low land)	13512	67.0
Weyna Dega (mid land)	2864	14.2
Dega (high land)	3785	18.8

### Mortality rates

#### Overall description of un-weighted mortality

**Fig. 2:**
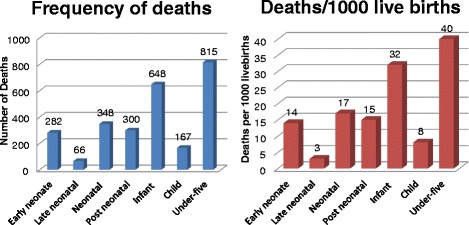
Number of deaths and mortality rate by age category, Gamo Gofa Zone, 2014

As depicted in Fig. 2, out of 20,161 children identified through the census of the 22 kebeles and the Arba Minch DSS, 815 died before their fifth birth day, providing an overall un-weighted under five mortality of 40/1000 live births. Of those, 282 died with in the first 7 days of birth, giving an un-weighted early neonatal mortality rate of 14/1000 live births. Sixty six of the children died after 7 days but within one month of age, giving an un-weighted late neonatal mortality rate of 3/1000 live birth. Accordingly, overall un-weighted neonatal mortality (early plus late neonatal) was 17/1000 live births. Three hundred of the children died after one month but before their first birth day, giving un-weighted post neonatal mortality rate of 15/1000 live births. So, the overall un-weighted infant mortality (neonatal plus post neonatal) was 32/1000 live births. Besides, 167 of the children died after their first birth date but before their fifth birth date, giving un-weighted child mortality rate of 8/1000 live birth (Fig. 2).

It is evident from Fig. 2, that about 79 and 44 % of all under-five mortalities occurred before their first birth date and within the first one month of age, respectively. About 82 % of neonatal deaths occurred within the first 7 days of life (Fig. 2).

As displayed in Table 2, over all under-five mortality was significantly low in DSS kebeles (32/1000 live birth) and urban kebeles (34/1000 live birth) than in non-DSS rural kebeles (52/1000 live birth) of Arba Minch district. Infant and neonatal mortalities were also significantly high in non-DSS kebeles of Arba Minch Zuria district than DSS and urban kebeles (Table 2).

**Table 2:** Mortality rates by: urban-rural, 3 categories of kebeles and sex of the child, Gamo Gofa Zone, 2014

Age category	By the three categories of kebeles (Un-weighted data)	Mortality/1000 Live births	[95 % Conf. Interval]		*P*-value
Under-five	Non-DSS Rural	52.24	47.30	57.18	1
	Urban	33.77	29.10	38.44	0.001
	DSS	32.30	28.04	36.56	0.001
Neonatal	Non-DSS Rural	25.54	22.04	29.05	1
	Urban	13.23	10.27	16.18	0.001
	DSS	11.02	8.50	13.53	0.001
Infant	Non-DSS Rural	41.20	36.79	45.62	1
	Urban	27.15	22.95	31.36	0.001
	DSS	25.81	21.99	29.63	0.001
Child	Non-DSS Rural	11.04	8.72	13.36	1
	Urban	6.61	4.52	8.71	0.001
	DSS	6.49	4.56	8.42	0.001
Age category	By rural–urban (Weighted data)	Mortality/1000 Live births	[95 % Conf. Interval]		*P*-value
Under-five	Over all	42.75	39.55	45.96	
	Rural	43.08	39.76	46.39	1
	Urban	33.77	29.10	38.44	0.003
Early Neonatal	Over all	15.23	13.28	17.17	
	Rural	15.40	13.39	17.41	1
	Urban	10.44	7.82	13.07	0.007
Late neonatal	Over all	3.44	2.51	4.37	
	Rural	3.47	2.51	4.43	1
	Urban	2.79	1.42	4.15	0.444
Neonatal	Over all	18.67	16.52	20.81	
	Rural	18.87	16.65	21.09	1
	Urban	13.23	10.27	16.18	0.006
Post neonatal	Over all	15.21	13.28	17.15	
	Rural	15.26	13.26	17.26	1
	Urban	13.93	10.90	16.96	0.480
Infant	Over all	33.88	31.02	36.75	
	Rural	34.13	31.17	37.09	1
	Urban	27.15	22.95	31.36	0.011
Child	Over all	8.87	7.38	10.35	
	Rural	8.95	7.41	10.49	1
	Urban	6.61	4.52	8.71	0.100
Age category	By Sex of the children (Weighted data)	Mortality/1000 Live births	[95 % Conf. Interval]		*P*-value
Under-five	Male	49.08	44.54	54.06	1
	Female	35.92	31.92	40.41	0.001
Infant	Male	39.50	35.43	44.01	1
	Female	27.83	24.32	31.83	0.001
Neonatal	Male	24.15	20.99	27.78	1
	Female	12.76	10.44	15.59	0.001

### Description of weighted mortalities

In order to account for the non-proportional allocation of the kebeles/study subjects among urban and rural, a weighted analysis was performed as explained at the analysis part of the method above. As indicated in Table 2, the overall weighted under-five mortality with its 95 % confidence interval was 42.75 (39.55-45.96) per 1000 live births. The corresponding weighted mortalities per 1000 live births with their corresponding 95 % confidence interval were 8.87 (7.38-10.35) for child, 33.88 (31.02-36.75) for infant, 15.21 (13.28-17.15) for post neonatal, 18.67(16.52-20.81) for neonatal, 3.44 (2.51-4.37) for late neonatal and 15.23 (13.28-17.17) for early neonatal (Table 2).

Significant difference of mortality was observed among rural and urban children. Under-five mortality was found to be significantly higher among rural kebeles (death/1000 live birth and 95 % confidence interval (CI) of 43.08 (39.76-46.39)) than urban kebeles (death/1000 live birth and 95 % CI of 33.77 (29.10-38.44)). Neonatal mortality was also high in rural kebeles (death/1000 live birth and 95 % CI of 18.87 (16.65-21.09)) than urban kebeles (death/1000 live birth and 95 % CI of 13.23 (10.27-16.18)) (Table 2).

There was significant difference of mortality among males and females. Under-five mortality was significantly high among males (death/1000 live birth and 95 % CI of 49.08 (44.54-54.06) thane females (death/1000 live birth and 95 % CI of 35.92 (31.92-40.41). Similarly, significantly high infant and neonatal mortality rates were observed among males than female (Table 2).

### Trends of mortality

**Fig. 3:**
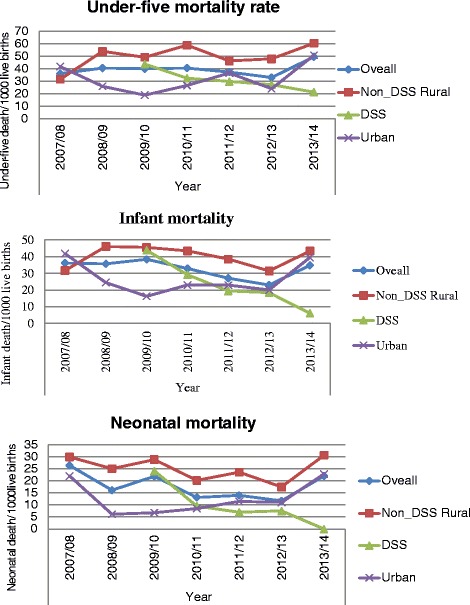
Trends of mortalities by different categories of the kebeles, Gamo Gofa Zone, 2014

In order to have full year mortality data to assess trends in child mortality, the data were reorganized in the Ethiopian calendar years (the calendar year starts at September). As displayed in Fig. 3, the result didn’t show a significant change in under-five mortality throughout the study time (*X*^2^ = 0.75, *p*-value = 0.39) in overall study kebeles. However, unlike other kebeles, under-five mortality in DSS kebeles found to be significantly decreasing (*X*^2^ = 10.16, *P* = 0.001). More or less similar trends were observed in infant and neonatal mortalities, i.e., fluctuating trends in the overall and non-DSS rural and urban kebeles but sharp reduction in DSS kebeles (Fig. 3).

## Discussion

The overall weighted under five, child, infant, post neonatal and neonatal mortalities were calculated to be 42.76, 8.87, 33.89, 15.22 and 18.68 per 1000 live births, respectively. These figures are lower than the national and regional reports of the latest EDHS 2011report [3]. The mortality rates identified by the current study are also lower than other pocket studies conducted in other parts of the country [4–8]. This may be because of socio-cultural differences in child caring and feeding practices of study populations. This was reflected by low prevalence of malnutrition (which is one of the leading causes of under-five mortality) in the study area (Zone) of the current study [11, 12]. The other reason may be due to time variation between the studies, as potential health service coverage has increased dramatically in the country in recent years. This was demonstrated by the current study that there were 100 % coverage of at least one health post and one health extension worker operating in the study kebeles of the current study and almost all the kebeles had access of health centers’ service within 10kms distance. Though, the other studies were basing on only few DSS kebeles (unlike the current study which covered larger population including DSS kebeles), most of them were either based on retrospective birth experiences of mothers [5–7] or prospective data of DSS kebeles [4, 8]. However, still there might be under reporting of deaths (survivor selection bias) in the current study as most of the data were collected retrospectively in a longer duration (within the past seven years before the survey).

About 79 and 44 % of all under-five mortalities occurred before their first birth date and within the first one month of age, respectively. Relatively, similar trend was observed in the 2011 EDHS report [3], in which 67 and 42 % of all under-five mortalities occurred before their first birth date and within the first one month of age, respectively. The occurrence of high mortality especially during post neonatal period might be attributed to infection mainly diarrheal disease, since this is the time when supplementary foods are started (given the poor hygienic condition of rural population of developing countries).

On the other hand, about 82 % of neonatal deaths occurred within the first 7 days of life. This was in spite of the above mentioned high potential health coverage of the study kebeles. It may be because of poor quality of maternal and child health services provided to the community, as health service delivery systems in developing countries have been criticized for failing to bring expected results at expected level, partly because of poor quality [13]. All these may signify importance of investigating quality of health services to improve and strengthen maternal and child health interventions during pregnancy, during and immediately after birth (through antenatal care (ANC), skilled birth attendance and early post natal care). This may help to avert majority of neonatal mortalities, as early neonatal mortalities are mainly caused by pregnancy and child birth related problems; including birth asphyxia, prematurity, maternal hypertension and obstetric hemorrhage [14–16].

Significantly, higher rate of under-five mortality was encountered among rural kebeles than in urban kebeles. Similar observation was found in the EDHS 2011 [3] report. This may be due to the relatively better access to health services and utilization of the services by urban population as a result of a relatively better awareness of the benefit of the health service.

Similar to the EDHS 2011 report [3], under-five mortality rate was significantly higher among males than in females in the current study. Similar finding was observed in a study which analyzed DHS data from sub Saharan African countries including Ethiopia’s EDHS 2011 data [17]. This may be owing to biologic differences of the two sexes, as genetic factors were reported to be reasons for higher mortality among males than females [18–20].

Unlike the other kebeles, under-five mortality significantly decreased in DSS kebeles during the study period. More or less similar trends were observed in infant and neonatal mortality, i.e., fluctuating trends in the overall and in non-DSS rural and urban kebeles but sharp reduction in DSS kebeles. Relatively low under-five mortality was also observed in DSS kebeles than non-DSS kebeles of the Arba Minch Zuria District and urban kebeles. This may be due to effect of frequent contact of data collectors, supervisors and researchers with the community which is not true in non-DSS sites. This might create more concern and motivation of HEWs and other health cadres working in the kebeles, because they knew mortalities are continuously monitored by the Project. Or it might create awareness about service utilization and child care in the communities owing to frequent visiting and questioning of the households to fill the questionnaires by data collectors of the Project. Previous study in India, revealed that, health education by visiting homes of the mothers had positive maternal behavior change that may positively affect child survival [21]. Similarly, frequent home visit by lay volunteers was shown to improve treatment outcome of tuberculosis in Iraq [22]. However, as DSS sites are becoming sources of evidence for magnitude and cause of mortalities in areas where vital event registrations are lacking (in Africa, Asia and Oceania) [23, 24], we suggest further investigation of whether such variations exist in other sites or not and the reasons of such variations.

Finally, this study covered a large number of populations from urban and rural kebeles and kebeles of different climatic/agro-ecological zones and DSS and non-DSS sites. However, the followings should be taken into consideration in interpreting the findings. There may be recall bias in determining date of birth and date of death, as most of the data were collected retrospectively. However, in majority cases we used the child’s immunization card. In the absence of immunization card we applied local calendars with the help of HEWs. There may be under reporting of deaths (survivor selection bias) especially for early child deaths, which may underestimate the rates. Some of the associations reported in this analysis may be confounded by other factors.

## Conclusions

The overall under-five mortality of the study area was found to be 43 per 1000 live births. The under-five mortality in the study area was lower than the national and regional reports. As significant numbers of children are dying during their early days of life in spite of high potential health coverage, investigation of quality of health services and strengthening of maternal and child health interventions during pregnancy, during and immediately after birth may help to avert majorities of neonatal mortalities. The mortality rates are significantly higher among rural communities than their urban counterparts. Therefore, child health interventions should give due attention, especially to those areas with low coverage of child and maternal health services. Finally, in order to address factors contributing for the continued risk of under-five mortality, study identifying the independent contributors of under-five mortality in the area need to be conducted. Besides, the actual reason for the relatively low rate of childhood mortality in DSS kebeles should be explored.

## Abbreviations

ANC: antenatal care
CI: confidence interval
DSS: demographic surveillance site
EDHS: Ethiopian demographic and health survey
HEWs: health extension workers
MDGs: Millennium development goals
SNNPR: Southern nations, nationalities and people’s region
WHO: World Health Organization
